# Mass Concentration, Source and Health Risk Assessment of Volatile Organic Compounds in Nine Cities of Northeast China

**DOI:** 10.3390/ijerph19084915

**Published:** 2022-04-18

**Authors:** Jianwu Shi, Yuzhai Bao, Liang Ren, Yuanqi Chen, Zhipeng Bai, Xinyu Han

**Affiliations:** 1 Faculty of Environmental Science and Engineering, Kunming University of Science and Technology, Kunming 650500, China; shijianwu@kust.edu.cn (J.S.); 20192207012@stu.kust.edu.cn (Y.B.); renliang@stu.kust.edu.cn (L.R.); 2 National-Regional Engineering Center for Recovery of Waste Gases from Metallurgical and Chemical Industries, Kunming 650500, China; 3 Faculty of Civil Engineering and Mechanics, Kunming University of Science and Technology, Kunming 650500, China; 20162210054@stu.kust.edu.cn; 4 State Key Laboratory of Environmental Criteria and Risk Assessment, Chinese Research Academy of Environmental Sciences, Beijing 100012, China; baizp@craes.org.cn

**Keywords:** VOCs, ozone formation potential, source apportionment, principal component analysis, health risk

## Abstract

From April 2008 to July 2009, ambient measurements of 58 volatile organic compounds (VOCs), including alkanes, alkenes, and aromatics, were conducted in nine industrial cities (Shenyang, Fushun, Changchun, Jilin, Harbin, Daqing, Huludao, Anshan and Tianjin) of the Northeast Region, China (NRC). Daqing had the highest concentration of VOCs (519.68 ± 309.88 μg/m^3^), followed by Changchun (345.01 ± 170.52 μg/m^3^), Harbin (231.14 ± 46.69 μg/m^3^), Jilin (221.63 ± 34.32 μg/m^3^), Huludao (195.92 ± 103.26 μg/m^3^), Fushun (135.43 ± 46.01 μg/m^3^), Anshan (109.68 ± 23.27 μg/m^3^), Tianjin (104.31 ± 46.04 μg/m^3^), Shenyang (75.2 ± 40.09 μg/m^3^). Alkanes constituted the largest percentage (>40%) in concentrations of the quantified VOCs in NRC, and the exception was Tianjin dominated by aromatics (about 52.34%). Although alkanes were the most abundant VOCs at the cities, the most important VOCs contributing to ozone formation potential (OFP) were alkenes and aromatics. Changchun had the highest OFP (537.3 μg/m^3^), Tianjin had the lowest OFP (111.7 μg/m^3^). The main active species contributing to OFP in the nine cities were C2~C6 alkanes, C7~C8 aromatic hydrocarbons, individual cities (Daqing) contained n-hexane, propane and other alkane species. Correlation between individual hydrocarbons, B/T ratio and principal component analysis model (PCA) were deployed to explore the source contributions. The results showed that the source of vehicle exhausts was one of the primary sources of VOCs in all nine cities. Additionally, individual cities, such as Daqing, petrochemical industry was founded to be an important source of VOCs. The results gained from this study provided a large of useful information for better understanding the characteristics and sources of ambient VOCs incities of NRC. The non-carcinogenic risk values of the nine cities were within the safe range recognized by the U.S. Environmental Protection Agency (HQ < 1), and the lifetime carcinogenic risk values of benzene were 3.82 × 10^−5^~1.28 × 10^−4^, which were higher than the safety range specified by the US Environmental Protection Agency (R < 1.00 × 10^−6^). The results of risk values indicated that there was a risk of cancer in these cities.

## 1. Introduction

In recent years, with sustained and rapid development of China’s national economy, industrialization and urbanization have been accelerating and the number of vehicles has increased dramatically. Energy consumption has been so significant that air pollution exceeded the environmental carrying capacity, with such pollution in cities and urban agglomerations. This is mainly manifested in the decline of atmospheric visibility and frequent occurrence of heavy pollution episode, such as urban photochemical smog. The importance and role of VOCs on the formation of troposphere ozone (O_3_) is well known [1,2,3]. Briefly, O_3_ is produced in the troposphere by the oxidation of VOCs in the presence of sunlight and nitrogen oxides. O_3_ is a recognized greenhouse gas [4] and is unhealthful at enhanced concentration [5]. VOCs, nitrogen oxides and hydroxyl radical are some important precursors of the troposphere O_3_, which is the root cause of urban photochemical smog [6]; Oxygenated volatile organic compounds in the atmosphere are further oxidized to form organic acids or secondary organic aerosols [7]. In some environmental problems, such as secondary organic pollution or human exposure to health effects, these VOCs have a significant impact. Additionally, VOCs can directly or indirectly affect human health. Studies have shown that VOCs are toxic, even carcinogenic [8,9].

Fossil fuel combustion of industrial activities had always been considered the major source of VOCs [10,11]. Lots of investigations have been undertaken for VOCs emissions from vehicle [12,13], coal-fired [14,15], biomass burning [16,17], solvent [17,18,19], petrochemical [20,21] and refinery [22,23].

**Figure 1:**
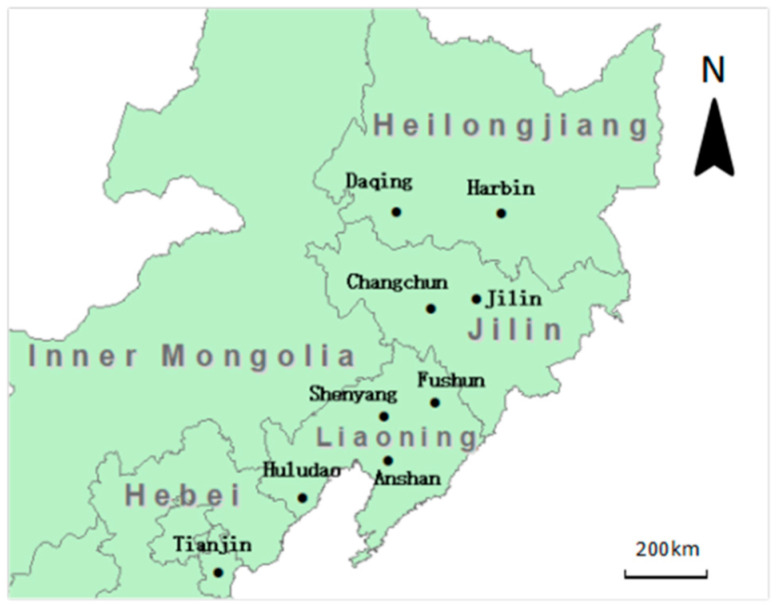
Nine sampling cities for whole air VOCs samples collected in Northeast Region of China in July 2009.

The NRC encompasses three big northeastern provinces, Liaoning, Jilin, Heilongjiang and the east area of Inner Mongolia (Figure 1). This region is bordered by Russia, Mongolia and North Korea to the north, northwest and southeast, respectively. It covers an area of 1,250,000 km^2^ and occupies 13% of the national total land area, with a population of about 120 million. NRC has the earliest heavy industry foundations in China, characterized by resource-reliant leading industries including iron/steel, coal, crude oil, machinery, metallurgy and chemical production. Shenyang belongs to Liaoning province which is a main city of the coastline-Bohai sea economic cycle. Tianjin is a hub of the coastline-Bohai sea economic circle, and it is an important traditional industrial base in China, whose industrial structure is similar to the cities in northeast region. Tianjin act as a hub of the Beijing-Tianjin-Hebei economic circle, which developed new industries rapidly, including petrochemical, equipment manufacturing, electronic information, bio-medicine, new energy, new materials, defense industry and other industries, and its car ownership, transport industry developed, causing it to be the same as the cities of the northeast region with higher VOCs emissions. As an important producer of energy and raw material, NRC and Tianjin play an important role in the national economic development and industrialization process. However, the traditional economic growth mode not only depletes the unreproducible natural resources but also causes severe environmental problems including deteriorated air, water and soil quality, which poses a significant health risk for people both within this region and in adjacent areas.

In this study, an atmospheric VOCs survey in nine cities was characterized, and intensive field campaigns were performed from April 2008 to July 2009 at sites located in nine cities, namely, Shenyang, Fushun, Changchun, Jilin, Harbin, Daqing, Huludao, Anshan, Tianjin. Major contributing sources were identified and quantified using the multivariate method, and the health risk of VOCs in nine cities was evaluated to provide a basis for improving the ambient air quality of NRC, which accumulated relevant basic data support the research on the change in China’s VOCs in recent years. This information is vital in terms of pollution control and risk management.

## 2. Methodology

### 2.1. Sampling Program

Most of the cities in the region are rich in mineral resources; nearly one-third of the typical resource cities in China are located in the northeast region. The location and information of the nine typical industrial cities examined in this study were shown in Figure 1 and Table 1, except Fushun and Jilin are located in the mountainous area, most of the studied areas are flat plains. All cities are in the mid-latitude temperate zone, which experience a continental monsoon climate, characterized by a long and chilly winter (about five to six month) when coal was heavily used for heating purposes.

Ambient samples were collected in 3.2 L fused silica-lined stainless steels canisters (Entech Instrument, Inc., Simi Valley, CA, USA) simultaneously in 9 cities of NRC, as shown in Figure 1. The sampling sites were selected to collect ambient samples away from the immediate influence of localized pollution sources, such as roadside vehicle exhausts and industrial emissions. Sufficient separation for the generally long-lived VOCs considered here was achieved using sites located at the tops of buildings, about 15 m high, all of these sites belonged to the air quality monitoring stations established by the local government environmental monitoring center. The sites were easily accessible for canister sampling and were located at an appropriate distance to avoid direct smoke stack emission or other industrial outputs nearby, which ensured the samples were representative of well-mixed air.

A sampling event was conducted in August 2009 in 9 cities. The air quality monitoring campaign represents designed to gain a better understanding of how ground-level ozone was formed and to determine the sources of VOC in the region of northeast China. Another sampling event was conducted in April, July and October 2008, and January 2009 at Shenyang and Tianjin, representing spring, summer, and autumn, winter, respectively. Two of them—Tianjin and Shenyang—were major metropolitan in NRC, at which five daily whole air sample canisters were each collected, five sites at Shenyang and six sites at Tianjin, during April, July and October 2008 and January 2009. The Tianjin and Shenyang were thought to be representative of the major metropolitan emission sites, respectively. The sampling dates were selected based on a forecast of meteorological conditions, each event lasted eight to ten consecutive days, and the weather during the sampling days was generally fine and the wind speeds calm as predicted by National Weather Service, favoring pollutant accumulation and interaction. Periods with such weather conditions were deliberately sought to maximize VOC signals and increase the likelihood of successful identification of sources using PCA. To sub-atmospheric sampling was used, the evacuated canister (b20 motor) was used, which was placed on the “roof platform” for 1 h sampling. No sources (e.g., air conditioning systems) immediately influenced the sampling. The flow rate is about 0.9 mL/s. A pressure gauge was used to check the completion of each sample collection (atmospheric pressure). 

### 2.2. Sampling Analysis

VOC analysis procedures followed the US Environmental Protection Agency (US EPA) methods TO-15. An 800-mL aliquot of air sample from each canister was concentrated using a three-stage cryofocusing pre-concentration system (Entech 7100, Entech Instruments, Simi Valley, CA, USA). The VOCs were separated on a capillary column (DB-624, 60 m × 0.25 mm × 1.4 µm) and quantified with a positive ionization detector. First, ambient air samples were pumped into the pre-concentrator, which has 3-stage cryotraps (Module 1 to Module 3). VOC compounds were initially trapped cryogenic on glass beads of Module 1 at −150 degrees Celsius by liquid nitrogen, then they were recovered by desorbing at 10 degrees Celsius to leave most of the liquid H_2_O behind in the first trap. The second cryotrap, which contains Tenax, was cooled to −30 degrees Celsius, which allows the trapping of VOCs while letting CO_2_ pass through. From Module 2, VOCs were back-flushed at 190 degrees Celsius and then focused again at −160 degrees Celsius in the Module 3 trap. The Module 3 trap then was rapidly heated to 60 degrees Celsius for 30 s. Helium was used as the purge gas for the cryogenic pre-concentrator and the carrier gas for the GC. Column DP-624 was initially held at 40 degrees Celsius for 3 min, then was raised to 90 degrees Celsius at a rate of 8 degrees Celsius each minute; then to 200 degrees Celsius at a rate of 6 degrees Celsius each minute, and finally was held for 9 min. Standard gas mixtures were VOC calibrating gas (1.0 ppm (parts per million), including 64 compounds, Spectra Gases To-15 (Spectra Gases Inc., Branchburg, NJ, USA)), PAM calibrating gas (1.0 ppm, including 57 compounds, Spectra Gases To-15) and 4-bromofluorobenzene (1.0 ppm, Spectra Gases To-15).

For the quality assurance and quality control, a laboratory blank sample was processed for ten samples with the same procedure as the real samples and no detectable VOCs were present. Field blanks were treated and no significant contamination was found. The total recovery efficiencies of VOCs ranged from 70% to 130% and the variance coefficient was less than 20%. Relative correlation for standard cure (standard gas including three concentration grads: 1 ppb (parts per billion), 2.5 ppb and 5 ppb) was in the range of 0.998–1.000 and the relative standard deviations of the response factor ranged from 2% to 25%.

The analyzed 58 VOC species from sampling could be categorized into 3 groups, including alkanes, alkenes (included acetylene) and aromatics, as shown in Table S1.

### 2.3. Ozone Formation Potential (OFP)

The maximum incremental reactivity (MIR) method can be used to identify species of highly reactive VOCs, and it was widely used in scientific studies [28]. To effectively control the VOCs in the atmospheric environment, the MIR method was used to determine the active components and key species in VOCs. The formula is as follows:

(1) $$OFP_{i}=\overset{n}{\underset{j=0}{\sum }}C_{ji}\times MIR_{j}$$

where *C_ji_* is the mass contribution of species *j* from source *i* (μg/m^3^) and *MIR_j_* is the MIR value of species *j*, as proposed by Carter [29].

### 2.4. Principal Component Analysis (PCA)

PCA can provide a more transparent view of the accumulated information. Factor analysis attempts to identify underlying variables, or factors, that explain the pattern of correlations within a set of observed variables. PCA is often used in data reduction to identify a small number of factors that explain most of the variance observed in a much larger number of manifest variables. In this study, PCA was carried out on selected 58 VOCs. The next step was to rotate the initial factor matrix. The rotation phase of factor analysis attempt to transform the initial matrix into one that was easier to interpret [30]. The varimax rotation of the matrix was selected which attempts to minimize the number of VOCs that have high loadings on a factor, this enhanced the interpretability of the factors. Only the principal components that explained more than 5% of the total variance of the data set were used as factors, factor loading determined the more representative species in each factor.

### 2.5. Health Risk Assessment

In this study, health risk assessment method was based on new methods of health evaluation inhaled route for a particular pollutant place in 2009 proposed by the US EPA (EPA-540-R-070-002), calculated as follows:

(2) $$EC_{i}=\frac{\rho \left(CA_{i}\right)\times ET\times EF\times ED}{AT}$$

(3) $$HQ=\frac{\rho \left(EC_{i}\right)}{\rho \left(RfC_{i}\right)\times 1000}$$

(4) $$R=\rho \left(EC_{i}\right)\times \rho \left(IUR_{i}\right)$$

(5) $$HI=\overset{n}{\underset{i}{\sum }}HQ_{i}$$

In the formula, *EC_i_* is the exposure concentration of VOCs*_i_*, the unit is μg/m^3^; *ρ*(*CA_i_*) is the environmental concentration of VOCs*_i_*, the unit is μg/m^3^; *ET* is the exposure time, the value is 24, the unit is h/d; *EF* is Exposure frequency, the value is 365, the unit is d/a; *ED* is the exposure time, the value is 70, the unit is a; *AT* is the average time, the unit is h, the value is 70 × 365 × 24; *HQ* is the non-carcinogenic risk Hazard quotient, *ρ*(*RfC_i_*) is the reference concentration of VOCs*_i_*, the unit is μg/m^3^; *R* is the lifetime carcinogenic risk value, *ρ*(*IUR_i_*) is the unit inhalation risk of VOCs*_i_* [31], the unit is (μg/m^3^)^−1^; *HI* is the hazard index.

## 3. Results and Discussion

### 3.1. Characteristics of Ambient VOCs Concentrations

#### 3.1.1. Spatial Characteristics of Ambient VOCs

**Figure 2:**
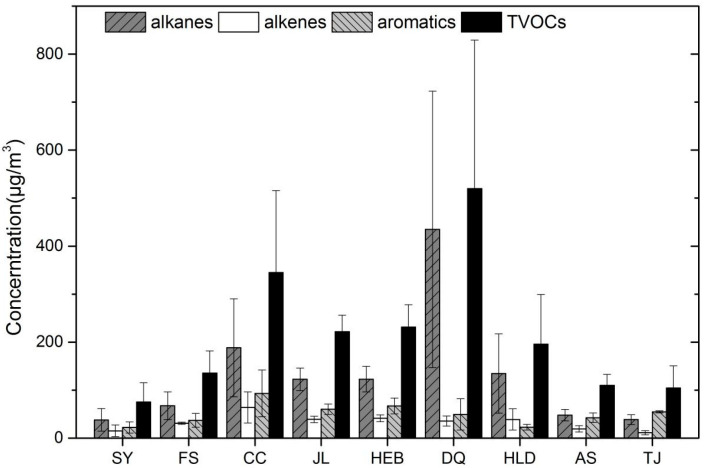
Concentrations of VOCs groups (alkane, alkene and aromatics) measured in nine cities of NRC in July 2009. SY: Shenyang, FS: Fushun, CC: Changchun, JL: Jilin, HEB: Harbin, DQ: Daqing, HLD: Huludao, AS: Anshan, TJ: Tianjin.

Figure 2 shows the VOCs in the group of alkanes, alkenes, alkynes, aromatics and aldehydes, the concentration and proportion in nine cities of NRC (Shenyang, Fushun, Changchun, Jilin, Harbin, Daqing, Huludao, Anshan and Tianjin) were discussed. Comparisons between these groups will help to understand the characteristics of VOCs in these urban areas. During the sampling event, high-temperature average of 30 degrees Celsius, VOCs rapidly volatilized into the atmosphere. As shown in Figure 2, in the nine cities, isoprene, as a natural source characteristic substance, accounted for less than 1%, the VOCs was released from anthropocentric emissions. the concentration of total VOCs (TVOCs) of Daqing (519.68 ± 309.88 μg/m^3^) was significantly higher than that in the other urban areas, 5 times higher than Shenyang (75.20 ± 40.09 μg/m^3^). Large fractions of aromatic compounds, especially toluene, were observed in nine cities. There had similar levels of light alkanes, as well as rich industries, which might explain the similarities. They had a high concentration of propane, likely due to the widespread domestic and vehicular use of liquefied petroleum gas. High levels of acetylene, toluene, ethylene, and ethane at this site probably originated from several anthropocentric sources such as vehicle exhaust, petrochemical industries, and industrial uses of solvents. Vehicular emissions were clearly identifiable from the significant levels of isobutane, isopentane, and benzene. 

It can be seen from Figure 2, alkanes were the highest contributors in the city of Daqing (83.69%), Shenyang (50.53%), Fushun (48.82%), Changchun (54.44%), Jilin (55.32%), Harbin (53.13%), Huludao (68.70%) and Anshan (43.75%), which come from the oil industry, the most important production in NRC. Daqing was oil city in extracting and refining, with the alkanes was 3–13 time than other cities, because of plenty of oil extraction, refining, fine chemicals products at Daqing. The contribution of the alkanes was only 36.92% at Tianjin, aromatics (52.35%) was the highest, meanwhile aromatics was the main contributors yet in Shenyang, Fushun, Anshan, Harbin, Changchun, Jilin was 29.29%, 27.31%, 38.75%, 29.02%, 27.00% and 27.10%, respectively. Additionally, the aromatics of Huludao and Daqing were lower level, at 11.52% and 9.48%. The alkenes of each city were at the lower level. Tianjin as the economic center of the northern region was an important production base of variate industry, which leaded to the coal was heavily used for fuel materials, and a large number of aromatics were released. In the area of Changchun, this may be due to the combined effect of alkanes (54.44%), aromatics (27.00%). Changchun was located in the central area of the NRC. It was the center of petrochemical urban agglomeration, the urban motor vehicle possession was fully developed, and would be affected by the transmission in the surrounding cities. 

Table 2 shows the mean concentrations of TVOCs measured in five typical cities (Guangzhou, Xinken, Shanghai, Taiwan and Nagoya) as well as nine cities of NRC. These areas were selected because the measurement periods were at least one year (including the seasonal cycles, except Guangzhou and Xinken, its measurement period was October to November 2004), and because the measurements covered major species of all major chemical groups (alkanes, alkenes, alkynes and aromatics). These concentrations must be compared with caution owing to differences in several measurement factors, such as the sampling periods. Nevertheless, the comparison was helpful for understanding the characteristics of VOC concentrations in these city areas. the total concentration at Fushun, Huludao, Anshan and Tianjin (135.43 ± 46.01 μg/m3, 195.92 ± 103.26 μg/m^3^, 109.68 ± 23.27 μg/m^3^ and 104.31 ± 46.04 μg/m^3^) was similar to the corresponding concentrations in Guangzhou (176.12 μg/m^3^), Xinken (132.20 μg/m^3^) [32] and Shanghai [33] (150.07 μg/m^3^), these cities had major manufacture including electronics, communication, paper, garments and textiles, food, shoes, and plastic. Tianjin’s transport industry was significantly developed, named the cargo distribution center of China. Additionally, Anshan was the iron and steel product center of China, with the production transported all over the world. Just as Shanghai was the center of the Yangtze River Delta region of China. Daqing and Taiwan had the same concentrations (519.68 ± 309.88 μg/m^3^ and 547.4 μg/m^3^, [34]), Taiwan was similar to Daqing, because Taiwan also had many petrochemical industries. Shenyang and Nagoya had the same concentrations (75.20 ± 40.09 μg/m^3^ and 79.09 μg/m^3^ [35], because the two cities also were major megalopoleis (population over 2 million) in central. By comparing the concentrations of VOCs in Shenyang and Tianjin in 2009 and 2019, it was found that concentration of VOCs at Shenyang was 65.33 μg/m^3^, decreased 13.1% in 2019 [36], the concentration of VOCs at Tianjin was 48.9 μg/m^3^, decreased 53.1% significantly in 2019 [37].

#### 3.1.2. Seasonal Characteristics of Ambient VOCs of Shenyang and Tianjin

In this study, the atmospheric VOCs of Shenyang and Tianjin were mainly observed. Samples of daily variation of VOCs were collected in April, July, October 2008 and January, July 2009 and in urban areas, respectively, which were taken as representative samples of winter, spring, summer and autumn.

**Figure 3:**
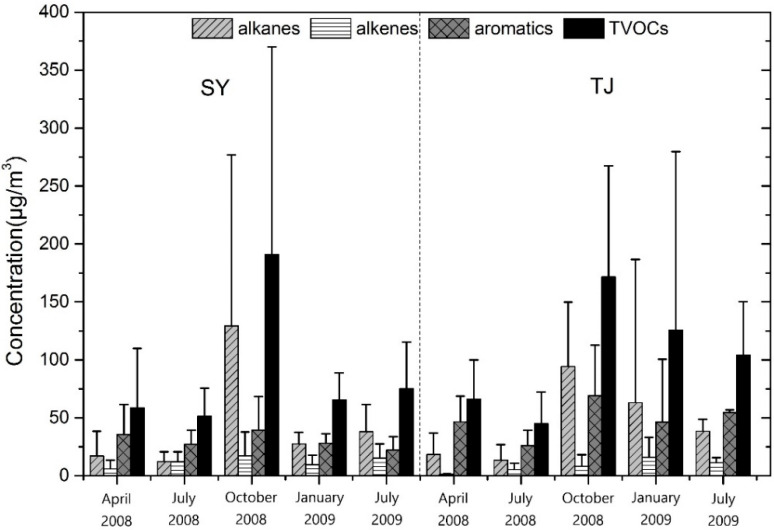
Concentrations of each chemical group (alkane, alkene, aromatics and TVOCs measured at Shenyang and Tianjin from 2008 to 2009. SY: Shenyang, TJ: Tianjin.

Figure 3 illustrates the average VOCs concentrations measured in each season for Shenyang and Tianjin. Average concentrations of TVOCs measured at the two cities showed significant seasonal variations. The VOCs concentrations in the heating season were about 2 to 4 times higher than those in non-heating seasons. The total amount of VOCs of Shenyang was higher in autumn than in other seasons (190.83 ± 179.28 μg/m^3^), and Tianjin was higher in winter (171.65 ± 95.96 μg/m^3^). The VOCs concentrations during the autumn and winter can be attributed to: (a) increased emissions from heating sources; (b) reduced photochemical degradation of some VOCs by solar radiation in winter. In contrast, the lowest concentrations in summer of two cities were attributable to the absence of seasonal sources (residential heating, cold start of vehicles), higher percentage in vapor phase, wash out effect, and photochemical degradation.

The low level of VOCs in spring and summer can likely be attributed to the climate characteristics of Shenyang and Tianjin were windy in spring and rainy in summer. Compared with spring and summer, the atmospheric stability and convection were weaker in autumn, due to the vertical mixing and diffusion of pollutants were reduced. In addition, the sunshine duration was significantly shortened in autumn, and the intensity of solar radiation and photochemical reaction decreased, leading to the degradation effect being weakened. so that the concentrations of VOCs reached their higher values in autumn. In winter, although January was the heating season, the fixed combustion emissions increased, which brought severe VOCs contamination.

Similar features were also founded for the average contributions of species groups across the four seasons for two cities (Figure 3). Aromatics were most abundant in spring and summer, with contributions ranging from 52.66~60.63% at Shenyang and 58.04~70.47% at Tianjin, followed by alkenes (23.91~29.17% and 27.72~30.10%). Alkanes increased in autumn, accounting for 67.79% and 54.95%, respectively. Whereas alkanes were highest at Tianjin in winter (50.33%), and alkanes and aromatics had the same level at Shenyang (42.02% and 43.25%). Alkenes were the lowest level all four seasons, with contributions ranging from 9.05~23.43% at Shenyang and 1.81~12.74%. Site and seasonal variations in VOCs concentrations were associated with several factors, such as emission strength, source fingerprint, meteorological conditions, and chemical losses at different sampling sites and seasons.

The observations for Tianjin were displayed during July 2008 and 2009 in Figure 3. The VOCs levels were significantly lower in 2008 (44.98 ± 27.35 μg/m^3^) than in 2009 (104.31 ± 46.04 μg/m^3^), it was 2 times. Three species groups also were significantly lower, and the proportion of the three VOCs groups were similar between 2008 and 2009. With the successful convening of the Olympic Games, Tianjin’s air quality reached international standards during the Olympic Games, indicated that the effective implementation of the Olympic security program played a key role. The concentration of Shenyang was similar between 2008 and 2009, indicated that the impact of implementation of the Olympic security program was small., because Shenyang was not the venue for the Olympic Games and far from Beijing, it had less impact on Beijing.

#### 3.1.3. Time Variation of VOCs at Shenyang and Fushun

**Figure 4:**
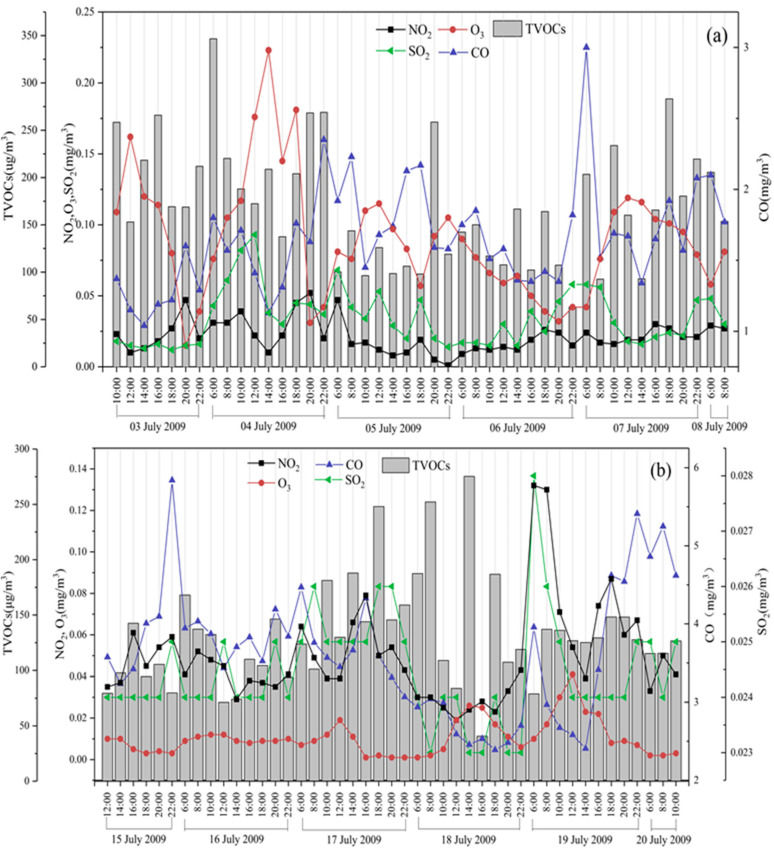
Time variation of measured NO_2_, O_3_, CO, SO_2_ and TVOCs at Shenyang (**a**) and Fushun (**b**) during the campaign.

Figure 4 displays the time variation of SO_2_, NO_2_, CO, O_3_ and VOCs observed at Shenyang. The observed VOCs enhancements (e.g., 4 and 7 July) were associated with highly elevated SO_2_, NO_2_ and CO, with ozone concentrations showed an opposite trend to other substances. For Fushun, the SO_2_, NO_2_, CO, O_3_ and VOCs were associated same as Shenyang on 15 and 17 July, however the occurrence of high values on other dates was inconsistent, which may be related to the influence of adverse meteorological conditions. Overall, this suggested that the observed high levels of VOCs may be attributed to same sources or processes. In most cases, a clear relationship between these high ozone level and either VOCs levels or NO_2_ and CO levels was observed.

### 3.2. Evaluation of OFP

VOCs as a primary pollutant in the atmosphere, greatly impact the formation of O_3_ and secondary organic aerosols. The concentration and chemical reactivity of VOCs in the atmosphere of different cities were different. In order to effectively control the VOCs in the atmospheric environment, the MIR method was used to determine the active components and key species in VOCs.

Table 3 is the OFP of VOCs in nine cities of NRC in the summer of 2009. It listed the OFP of the top 10 VOCs and TVOCs and showed the OFP of TVOCs in Harbin, Daqing, Huludao, Changchun, Shenyang, Jilin, Anshan, Fushun and Tianjin were 360.00 μg/m^3^, 426.90 μg/m^3^, 262.60 μg/m^3^, 537.30 μg/m^3^, 121.60 μg/m^3^, 346.20 μg/m^3^, 175.70 μg/m^3^, 222.10 μg/m^3^ and 111.70 μg/m^3^. The highest OFP was 537.30 μg/m^3^ in Changchun, and the lowest is 111.70 μg/m^3^ in Tianjin.

The OFP of the top 10 species of VOCs in nine cities were dominated by alkenes and aromatics, and the top 10 species accounted for 60.1~76.5% of the OFP of TVOCs. The species of the highest OFP level in VOCs of nine cities as follows: toluene of Harbin, toluene of Daqing, propylene of Huludao, toluene of Changchun, 1,3-butadiene of Shenyang, toluene of Jilin, toluene of Anshan, ethylene of Fushun, toluene of Tianjin. Toluene and ethylene were the mainly active component of each city in the top 10 species of OFP. There were five kinds of alkanes in the top 10 species of OFP in VOCs in Daqing. The top 10 species of OFP at Tianjin and Anshan were mainly aromatic hydrocarbons. Therefore, the main active species in the NRC were C2~C6 alkanes, C7~C8 aromatic hydrocarbons, individual cities (Daqing) included n-hexane and propane and other alkane species.

**Figure 5:**
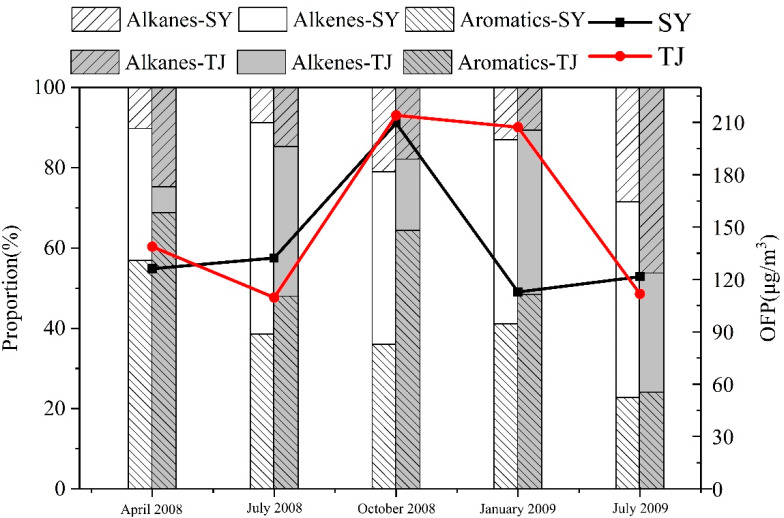
Seasonal variation of contributions to OFP by different VOC groups (alkanes, alkenes and aromatics) at Shenyang and Tianjin. SY: Shenyang, TJ: Tianjin.

Figure 5 lists the OFP of the TVOCs in four seasons at Shenyang and Tianjin. The highest of OFP was in autumn (208.65 μg/m^3^ at Shenyang and 213.03 μg/m^3^ at Tianjin), which was in line with the highest concentration of VOCs in autumn at the two cities. It showed that changes in the concentrations of VOCs had a certain impact on OFP. At Shenyang, the OFP of other seasons had not change much (112.24~131.35 μg/m^3^). At Tianjin, the followed by January 2009 (206.20 μg/m^3^), April 2008 (136.00 μg/m^3^), July 2008 (111.70 μg/m^3^), July 2008 (109.06 μg/m^3^). In January of Tianjin, the concentration of TVOCs was the lowest in four seasons, but the OFP was higher. The concentration of alkenes in January (19.69 μg/m^3^) was higher than other seasons (4.34~10.19 μg/m^3^), and the MIR of alkenes was higher than alkanes and aromatics. The MIR method was used to evaluate the contributions of VOC groups to ozone formation, indicated the amount of O_3_ increased for every 1 g added of hydrocarbons.

Additionally, Figure 5 shows the OFP of three comparisons of VOCs (alkanes, alkenes, aromatic) during the sampling periods in each of the seasons. In Shenyang, the contributions of alkenes to OFP accounted for the larger part in July, October 2008 and January, July 2009 (52.7%, 43.0%, 45.9% and 48.7%), and it was 32.9% in April 2008. Aromatic had the same contribution to ozone as alkenes (36.0~56.9%), especially in April 2008 (56.9%), in which the concentration of alkanes was a large part for VOCs at Shenyang (47.2%), the OFP was lower than alkenes and aromatic (8.8~28.5%). At Tianjin, the OFP of aromatic (48–68.8%) was highest than alkenes and alkanes, except July 2009 (24.1%). This indicated that aromatic were the main contributors to ozone at Tianjin. The alkenes were important role for OFP in July and January 2008 (37.3% and 40.9%). The same as Shenyang, the OFP of alkanes was the lowest at Tianjin. The result suggests that even though the measured concentrations of alkanes were the highest, their contributions to OFP were less than alkenes and aromatics, due to the lower hydroxyl radical reactivity. Compared OFP of July 2008 with July 2009 at Tianjin, although both of the total OFP was similar (109.66 μg/m^3^ and 111.7 μg/m^3^), the OFP of aromatics accounted for the main part in 2008 (47.96 μg/m^3^), and alkanes dominated in 2009 (46.24 μg/m^3^).

### 3.3. Source Apportionments of VOCs

#### 3.3.1. VOCs Sources Identification of Nine Cities in the Summer

Correlations between the individual hydrocarbons were used to identify different sources of VOCs. Ethyne is a characteristic product of combustion processes, whereas i-pentane is a marker for gasoline evaporation. In order to determine the impact of combustion and gasoline evaporation on VOCs concentrations, correlation coefficients (R^2^) of the different hydrocarbons with ethyne and i-pentane were calculated for the nine cities (Table S2).

In NRC, Short-chain alkenes (ethene, propene, 1-butene, i-butene and 1,3-butadiene) and aromatic compounds showed a good correlation with ethyne (R^2^ > 0.6) suggested that their primary source was combustion, except Huludao. The majority of aromatic compounds also correlated well with i-pentane indicated that gasoline evaporation was likely an additional source (Shenyang being an exception with a correlation coefficient of below 0.5). Finally, the good correlation of long-chain alkanes (C6~C10) with i-pentane indicated that solvent evaporation was more likely the main source for those gases.

In an attempt to differentiate between vehicular emissions and other combustion sources, the benzene to toluene ratio (B/T) was considered. Correlation and ratios between the BTEX (benzene, toluene, ethylbenzene and xylene). The B/T ratio in nine cities of NRC in this study ranged from 0.2 to 1.39 (Table S2). To identify the cities with a B/T ratio characteristic of vehicular emission, only cities with an average B/T ratio between 0.4 and 0.8 were considered. Shenyang, Anshan and Tianjin had a B/T ratio close to 0.6 (0.54, 0.41 and 0.44), suggested vehicular emissions as the primary source of VOCs in these cities. The abundance of the ethylbenzene and 1-butylene relative to the total non-methane hydrocarbons (NMHCs) was also significantly higher in the cities. In urban, the source of ethylbenzene and 1-butylene were relatively simple, mainly from traffic emissions [38]. The B/T ratio of Huludao between 0.6 and 1 (0.98), which showed it was affected by vehicle exhaust and coal combustion. The ratio of Fushun was higher than 1 indicated that had been reported for biofuel, charcoal and coal burning [39,40]. Additionally, the ratio of other cities (Changchun, Jilin, Harbin, Daqing) were lower than 0.4 (0.16, 0.2, 0.37 and 0.24), suggested these cities were affected by multiple pollution sources.

#### 3.3.2. VOCs Sources Identification at Shenyang and Tianjin in Four Seasons

Before applying PCA to the VOC data, the individual sample was inspected and those with unusually high or low concentrations were identified as possible outliers, the sensitivity analysis was conducted. The suspected outliers were removed from the dataset one at a time until a stable PCA result was achieved and further exclusion of samples in a random fashion had very little effect on the PCA outputs.

In total, 37 species at Shenyang and 44 species at Tianjin were chosen for PCA analysis, these were the most abundant compounds (accounting for 72.42% at Shenyang and 87.23% at Tianjin of the TVOCs) and efficient tracers for major VOCs sources. Five and six components were extracted from the application of PCA to the data of VOCs at Shenyang and Tianjin, respectively (Tables S3 and S4). Here, Kaiser’s criteria were adopted to decide the appropriate number of factors to be retained (only factors with eigenvalues > 1) [41]. Good model solutions were achieved when the fractions of pollutant variance explained by the PCA model were as close to one as possible, the high loading for some VOCs on more than one factor indicated that there was more than one source for these chemicals.

The components in Table S3 represented the following source categories: (1) Source of industrial fuel combustion; (2) Vehicle exhausts including internal engine combustion and unburned emissions such as evaporation of vehicle fuels from motors and vehicle fuel tanks; (3) Liquefied petroleum gas leakage or natural gas leakage from vehicle tanks, cooking; (4) Biogenic emissions; (5) Use of solvent. The Factor 1 explained 33.48% of the total variance, and the Factor 4 explained 5.11% of the total variance. The Factor 1, Factor 2, Factor 3 and Factor 4 explained 74.94% of the total variance.

Factor 1: High factor loading were found for ethyne, alkenes, alkanes and aromatics, species were so complex. Ethyne were associated with combustion, and the aromatics and alkanes were from industry combustion. The possible sources included industry combustion and vehicle exhausts.

Factor 2: 1,3-butadiene in the atmosphere was the characteristic product of internal combustion engines [38]. and propane were the main components of liquefied petroleum gas and they were also components of natural gas [42,43], high factor loadings of these compounds could attribute to the liquefied petroleum gas or natural gas leakage. In NRC, liquefied petroleum gas was the major fuel for cooking. Moreover, liquefied petroleum gas had been used as fuels in some vehicles such as taxis in recent years, where natural gas was used as fuel in power plants. Therefore, the factor was labeled as “vehicle exhausts”, including exhaust, running loss and fraction of resting loss emissions, and evaporate emissions from stationary vehicles.

Factor 3: I-pentane was a marker for gasoline evaporation. Additionally, ethylbenzene, phenylethylene and p,m,o-xylene had high factor loading in this factor. It was known that these species had the largest portion in solvents [44], which were the major constituent of paints. Species in Factor 3 could result from the evaporation of gasoline or solvents (such as paints).

Factor 4: Isoprene was one of the most reactive hydrocarbon species and was used as a tracer for biogenic emissions, the possible sources was biogenic emissions.

Factor 5: high factor loading was found for cyclohexane. Cyclohexane was often used as a solvent for rubber, paints, varnishes. Additionally, cyclohexane also was adhesive thinners, and oil extractants. The high factor loading was observed suggesting sources of solvent dominated the compound.

**Figure 6:**
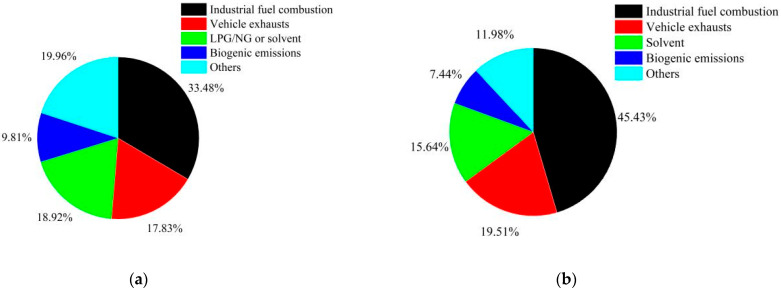
Source apportionment of VOCs at Shenyang (**a**) and Tianjin (**b**).

From the PCA-resolved result of VOCs at Shenyang (Figure 6a), the sources of combustion (included industry combustion and vehicle exhausts) contributed the most to ambient VOCs, at 33.48% of the total concentration, followed by sources of liquefied petroleum gas/natural gas or solvent (18.92%), vehicle exhausts (17.83%), biogenic emissions (9.81%).

The components in Table S4 represented the following source categories: (1) Source of industrial fuel combustion; (2) Vehicle exhausts (3) Biogenic emissions; (4) Use of solvent. The Factor 1 explained 33.48% of the total variance, and the Factor 5 explained 5.11% of the total variance. The Factor 1, Factor 2, Factor 3 and Factor 4 explained 74.94% of the total variance.

Factor 1: The high-carbon chain n-alkanes had high loadings, such as heptane, n-octane and n-pentane, and the proportion of n-alkanes of high-carbon chain in coal-burning gas was large. Although i-pentane was a marker for gasoline evaporation, but emitted during combustion processes. Therefore, the factor was label as “industrial fuel combustion”, respectively.

Factor 2 and Factor 6: In Factor 2, the vehicular internal engine combustion and the unburned vehicle emissions can be identified by high factor loading on propene, 1,3-butadiene and alkanes. In Factor 6, 1-butylene were relatively simple, mainly from traffic emissions. So species in Factor 2 and Factor 6 could result from vehicle emissions.

Factor 3 and Factor 5: In the two Factors, high factor loading was found for ethylbenzene, xylene isomers, phenylethylene and propylbenzene isomers. which indicated species could result from the evaporation of solvents, such as paints.

Factor 4: Isoprene had high loading and was used as a tracer for biogenic emissions. Thus, it was label as “biogenic emissions”.

From the PCA-resolved result of VOCs at Tianjin (Figure 6b), the sources of Industrial fuel combustion contributed the most to ambient VOCs, at 45.43% of the total concentration, followed by sources of vehicle exhausts (19.51%), use of solvent (15.64%), biogenic emissions (7.44%).

### 3.4. Health Risk Assessment

Benzene, toluene, ethylbenzene, p,m-xylene, and o-xylene are important VOCs species in the atmosphere. Table 4 shows the comparison of the non-carcinogenic and carcinogenic risks of benzene series in nine cities of NRC with other cities. The results showed that the non-carcinogenic risk values of Shenyang, Changchun, Huludao, and Tianjin were all expressed as benzene > p,m-xylene > o-xylene > ethylbenzene > toluene, whereas Fushun, Jilin, Harbin, Daqing and Anshan were expressed as benzene > p,m-xylene > o-xylene> toluene > ethylbenzene, ranging from 1.31 × 10^−3^~5.46 × 10^−1^, within the safety range recognized by the US Environmental Protection Agency (HQ < 1). The lifetime carcinogenic risk value of benzene was between 3.82 × 10^−5^~1.28 × 10^−4^, which was higher than the safety range stipulated by the US Environmental Protection Agency (R < 1.00 × 10^−6^), indicated that there was a risk of carcinogenesis, and the human body was exposed to the environment for a long time. It may induce immune diseases of the lymphatic system and leukemia, which should be paid more attention.

In this study, the lifetime carcinogenic risk quotient of benzene at Fushun, Jilin and Harbin was higher than Guangzhou and Beijing, whereas the lifetime carcinogenic risk quotient of benzene at Shenyang and Changchun was lower than that of Guangzhou and Beijing. Benzene had the highest non-carcinogenic risk quotient in the nine cities of NRC, whereas Guangzhou and Beijing had the highest non-carcinogenic risk quotient of toluene, and the HQ value toluene of Harbin (1.06 × 10^−2^) was higher than that in eight other cities in NRC. The HQ values of p,m-xylene at Harbin and Tianjin were higher than at Guangzhou, and lower in other cities. However, all the HQ values in nine cities were higher than at Beijing, indicated that other cities had a higher non-carcinogenic risk of p,m-xylene than Beijing. In general, the nine cities of NRC had higher health risks (higher HI values) than other cities. In particular, it should be noted that the carcinogenic risk of benzene in the region had exceeded the safety threshold. Therefore, it is necessary to decrease the emission of benzene, control and reduce their carcinogenic risk.

## 4. Conclusions

Ambient concentrations of VOCs were measured in nine cities of NRC. Daqing had the highest average concentrations, followed by Changchun, Harbin, Jilin, Tianjin, Fushun, Huludao, Anshan and Shenyang. The chemical patterns of VOCs were similar in nine cities, with alkanes and as the dominant group. However, the most important VOCs contributing to ozone formation potential (OFP) were alkenes and aromatics. For OFP of seasonal characteristics at Shenyang and Tianjin. This result suggested that even though the measured concentrations of alkanes were the highest, their contributions to OFP were less than alkenes and aromatics, due to the lower hydroxyl radical reactivity. 

Using B/T ratio of 0.6 ± 0.2, three groups of cities were identified: three cities (Shenyang, Anshan and Tianjin) with the ratio in the range of 0.4–0.8, Huludao with a ratio between 0.6–1 (0.98), four cities (Changchun, Jilin, Harbin and Daqing) with a ratio lower than 0.4. The B/T ratio of about 0.6 and the good correlation with i-pentane, suggested vehicular emissions (vehicular combustion and gasoline evaporation) were likely the main sources of the identified hydrocarbons for those cities. Combustion and vehicular emissions were still the primary source of non-methane hydrocarbons for the second group of cities. Lower ratio of the third group suggesting these cities affected multiple pollution sources. Four sources were identified for VOCs by the PCA model at Shenyang: (1) combustion (included industry combustion and vehicle exhausts); (2) sources of liquefied petroleum gas/natural gas or solvent; (3) vehicle exhausts; (4) biogenic emissions. And four sources were identified at Tianjin: (1) Industrial fuel combustion; (2) vehicle emissions; (3) use of solvent; (4) biogenic emissions.

The non-carcinogenic risk values of Shenyang, Fushun, Changchun, Jilin, Harbin, Daqing, Huludao, Anshan and Tianjin were within the safe range recognized by the U.S. Environmental Protection Agency (HQ < 1), and the lifetime carcinogenic risk value of benzene was 3.82 × 10^−5^~1.28 × 10^−4^, higher than the safety range specified by the US Environmental Protection Agency (R < 1.00 × 10^−6^), indicate that there is a risk of cancer. The HI values in nine cities of NRC were higher than other cities, and the health risk level was higher than that of other cities. 

This study provides a data base for the health risk assessment and ambient air quality in nine cities in NRC, and accumulates relevant basic data to support the research about the changes of VOCs in China in recent years. This information is crucial in pollution control and risk management.

## Figures and Tables

**Table 1:** Description of the nine cities examined in this study.

City	Location	Urban Area	Population	Main Industry	Sampling Size	DescriptionYear	References
Shenyang	41°11′–43°02′ N, 122°25′–123°48′ E	3945 km^2^	7.1 million	Equipment manufacturing, metal smelting, medical	145	2008	[24]
Fushun	41°41′–42°38′ N, 123°39′–125°28′ E	675 km^2^	2.2 million	Coal mining, petrochemical, fine chemicals, aluminum	45	2008	[24]
Anshan	40°27′–41°34′ N, 122°10′–123°13′ E	624.3 km^2^	3.5 million	Iron and steel, minerals processing	7	2008	[24]
Huludao	39°59′–41°12′ N, 119°12′–121°02′ E	2303 km^2^	2.8 million	Petrochemical, equipment manufacturing, zinc	7	2008	[24]
Changchun	43°05′–45°15′ N, 124°18′–127°02′ E	4906 km^2^	3.6 million	Automobile, medical, food, photoelectronic	7	2008	[25]
Jilin	42°31′–44°40′ N, 125°40′–127°56′ E	3636 km^2^	4.3 million	Petrochemical, metallurgy, automobile, carbon production	7	2008	[25]
Harbin	44°04′–46°40′ N, 125°42′–130°10′ E	7086 km^2^	9.5 million	Equipment manufacturing, medical, food, petrochemical	7	2008	[26]
Daqing	45°46′–46°55′ N, 124°19′–125°12′ E	5107 km^2^	2.8 million	Oil extraction, petrochemical	7	2008	[26]
Tianjin	38°33′–40°15′ N, 116°42′–118°04′ E	11,946 km^2^	11.7 million	Equipment manufacturing, petrochemical	108	2008	[27]

**Table 2:** Comparison of TVOCs concentrations (μg/m^3^) between cities of NRC and other cities.

City	Concentration		Time	References
	Ave	S.d		
SY	75.20	40.09	April 2008 to July 2009	this study
Fushun	135.43	46.01	July 2009	
Changchun	345.01	170.52	July 2009	
Jilin	221.63	34.32	July 2009	
Harbin	231.14	46.69	July 2009	
Daqing	519.68	309.88	July 2009	
Huludao	195.92	103.26	July 2009	
Anshan	109.68	23.27	July 2009	
Tianjin	104.31	46.04	April 2008 to July 2009	
Guangzhou	176.12	-	October to November 2004	[32]
Xinken	132.20	-	October to November 2004	[32]
Shanghai	150.07	-	July 2006 to February 2010	[33]
Taiwan	547.4	-	December 1998 to May 1999	[34]
Nagoya (Japan)	79.07	-	December 2003 to November 2004	[35]
Shenyang	65.33	-	All of 2019	[36]
Tianjin	48.90	-	All of 2019	[37]

“-” means there is no data in this reference.

**Table 3:** Top ten species of contributions to ozone formation (μg/m^3^) by different VOC species in nine cities of NRC.

Harbin	OFP	Daqing	OFP	Huludao	OFP
Toluene	53.09	Toluene	51.49	Propene	44.94
Propene	30.12	n-Hexane	36.56	1-Butylene	37.97
Ethene	29.95	1-Hexene	32.28	Trans-2-butene	20.94
1-Butylene	25.73	Propane	28.46	Ethene	15.72
Isoprene	25.11	n-Pentane	22.94	Propane	15.22
Trans-2-butene	17.17	1-Butylene	19.92	1-Pentene	14.37
n-Butane	16.95	Isopentane	18.62	Cis-2-butene	12.93
n-Hexane	12.84	Ethene	16.47	Toluene	12.10
Cis-2-butene	12.43	Isobutane	15.32	1-Hexene	9.68
p,m-Xylene	11.87	Isoprene	14.31	Trans-2-pentene	9.13
Accounting for TVOCs	0.653	Accounting for TVOCs	0.601	Accounting for TVOCs	0.735
TVOCs	360.00	TVOCs	426.90	TVOCs	262.60
**Changchun**	**OFP**	**Shenyang**	**OFP**	**Jilin**	**OFP**
Toluene	98.27	1,3-Butadiene	20.01	Toluene	51.42
1-Hexene	68.98	Toluene	14.17	1-Butylene	34.20
1-Butylene	50.73	1-Hexene	8.61	Propene	31.86
Trans-2-butene	43.70	Ethene	7.72	Ethene	31.55
n-Hexane	35.28	p,m-Xylene	6.08	n-Butane	22.57
n-Butane	33.41	1-Butylene	5.98	Trans-2-butene	17.72
Cis-2-butene	27.87	Propene	4.52	Isoprene	16.98
Ethene	20.98	1-Pentene	4.10	n-Hexane	13.58
Propene	17.30	Trans-2-butene	3.69	Cis-2-butene	12.84
p,m-Xylene	14.65	o-Xylene	3.67	p,m-Xylene	9.52
Accounting for TVOCs	0.765	Accounting for TVOCs	0.646	Accounting for TVOCs	0.700
TVOCs	537.30	TVOCs	121.60	TVOCs	346.20
**Anshan**	**OFP**	**Fushun**	**OFP**	**Tianjin**	**OFP**
Toluene	28.90	Ethene	24.05	Toluene	21.11
Ethene	22.9	Propene	22.08	Ethene	8.90
Propene	14.11	Isoprene	19.14	p,m-Xylene	7.90
1,2,3-Trimethylbenzene	11.52	1-Hexene	18.31	Propene	7.35
p,m-Xylene	8.23	Toluene	18.26	1-Butylene	5.03
n-Hexane	6.88	Isopentane	14.96	o-xylene	4.93
1-Butylene	6.86	1-Butylene	12.63	1,2,3-Trimethylbenzene	3.67
m-Ethyltoluene	6.49	Trans-2-butene	8.37	Isopentane	3.61
4-Ethyltoluene	5.60	Cis-2-butene	6.83	1,2,4-Trimethylbenzene	3.52
o-Xylene	5.44	n-Hexane	5.92	Ethylbenzene	3.35
Accounting for TVOCs	0.666	Accounting for TVOCs	0.678	Accounting for TVOCs	0.621
TVOCs	175.70	TVOCs	222.10	TVOCs	111.70

**Table 4:** Comparison of non-carcinogenic and carcinogenic risks of benzene series in nine cities of NRC with other cities.

City	R	HQ					HI	References
		Benzene	Toluene	Ethyl-Benzene	p,m-Xylene	o-Xylene		
Shenyang	3.82 × 10^−5^	1.63 × 10^−1^	2.15 × 10^−3^	2.67 × 10^−3^	1.94 × 10^−2^	1.30 × 10^−2^	2.00 × 10^−1^	this study
Fushun	6.57 × 10^−5^	2.81 × 10^−1^	5.12 × 10^−3^	3.76 × 10^−3^	2.77 × 10^−2^	2.12 × 10^−2^	3.39 × 10^−1^	
Changchun	4.18 × 10^−5^	1.78 × 10^−1^	1.48 × 10^−3^	1.99 × 10^−3^	1.58 × 10^−2^	1.00 × 10^−2^	2.08 × 10^−1^	
Jilin	1.28 × 10^−4^	5.46 × 10^−1^	1.68 × 10^−3^	1.43 × 10^−3^	1.17 × 10^−2^	1.14 × 10^−2^	5.72 × 10^−1^	
Harbin	6.81 × 10^−5^	2.91 × 10^−1^	1.06 × 10^−^^2^	9.53 × 10^−3^	4.23 × 10^−2^	2.82 × 10^−2^	3.82 × 10^−1^	
Daqing	4.26 × 10^−5^	1.82 × 10^−1^	5.55 × 10^−3^	4.19 × 10^−3^	2.74 × 10^−2^	2.08 × 10^−2^	2.40 × 10^−1^	
Huludao	4.98 × 10^−5^	2.13 × 10^−1^	1.31 × 10^−3^	2.38 × 10^−3^	1.26 × 10^−2^	1.09 × 10^−2^	2.40 × 10^−1^	
Anshan	5.01 × 10^−5^	2.14 × 10^−1^	3.12 × 10^−3^	2.73 × 10^−3^	2.37 × 10^−2^	1.56 × 10^−2^	2.59 × 10^−1^	
Tianjin	4.25 × 10^−5^	1.82 × 10^−1^	3.16 × 10^−3^	3.71 × 10^−3^	3.09 × 10^−2^	1.91 × 10^−2^	2.39 × 10^−1^	
Guangzhou	5.34 × 10^−5^	2.28 × 10^−1^	3.95 × 10^−1^	4.26 × 10^−3^	3.06 × 10^−2^	2.42 × 10^−2^	2.91 × 10^−1^	[31]
Beijing	4.19 × 10^−5^	1.57 × 10^−1^	2.39 × 10^−1^	3.29 × 10^−3^	8.06 × 10^−3^	3.53 × 10^−3^	1.96 × 10^−1^	[45]

R: the lifetime carcinogenic risk value, HQ: non-carcinogenic risk Hazard quotient, HI: hazard index.

## Data Availability

The data used in this paper can be provided by Jianwu Shi (shijianwu@kust.edu.cn).

## Supplementary Materials

The following supporting information can be downloaded at: https://www.mdpi.com/article/10.3390/ijerph19084915/s1, Table S1. Comparisons of the normal concentrations of each chemical group (alkane, alkene, aromatics) measured in nine cities of NRC in July 2009, Table S2. Correlation coefficient (R^2^) of the identified VOCs with ethyne and i-pentane for the nine cities sampled, and benzene to toluene ratio (B/T) in nine cities of NRC, Table S3. Source apportionment of VOCs at Shenyang by PCA, Table S4. Source apportionment of VOCs at Tianjin by PCA.

## Nomenclature

VOCs	volatile organic compounds
NRC	Northeast Region, China
OFP	ozone formation potential
PCA	principal component analysis model
TVOCs	total VOCs
R^2^	correlation coefficients
B/T	benzene to toluene ratio
BTEX	benzene, toluene, ethylbenzene and xylene
R	lifetime carcinogenic risk
HQ	non-carcinogenic risk Hazard quotient value
HI	hazard index
