# Supplementary material for: Mass Concentration, Source and Health Risk Assessment of Volatile Organic Compounds in Nine Cities of Northeast China
Source: Int J Environ Res Public Health. 2022 Apr 18;19(8):4915. doi: 10.3390/ijerph19084915 (PMC9028055; doi:10.3390/ijerph19084915)
Supplement: Supplementary file 1 [file ijerph-19-04915-s001.zip › ijerph-1649976-supplementary.pdf]

International Journal of
Environmental Research and Public Health

MDPI

Supplementary Materials

Mass Concentration, Source and Health Risk Assessment of Volatile Organic Compounds in 9 Cities of Northeast China

Supplementary Materials

Table S1. Comparisons of the normal concentrations of each chemical group (alkane, alkene, aromatics) measured in 9 cities of NRC in July, 2009.

Number	Species	Shenyang		Anshan		Huludao		Tianjin		Fushun		Changchun		Jilin		Harbin		Daqing	
		ave	s.d	ave	s.d	ave	s.d	ave	s.d	ave	s.d	ave	s.d	ave	s.d	ave	s.d	ave	s.d
1	n-Hexane	3.44	3.45	10.31	3.47	5.63	3.17	4.77	4.61	8.92	5.98	52.86	32.83	20.35	11.63	19.23	10.58	54.78	38.98
2	2,2,4-Trimethyl pentane	0.15	0.25	0.30	0.20	0.11	0.01	0.17	0.00	0.28	0.23	0.23	0.08	0.14	0.02	0.21	0.08	1.46	2.45
3	Cyclohexane	3.36	3.72	2.46	0.39	1.54	0.67	1.58	0.77	0.11	0.00	10.46	6.34	4.67	1.71	4.72	2.15	14.42	8.98
4	Heptane	0.84	0.80	0.87	0.39	3.06	1.35	0.97	0.29	1.24	0.77	0.89	0.34	0.73	0.21	1.43	0.35	12.15	8.90
5	n-Butane	1.42	1.26	1.49	0.41	5.85	3.77	1.12	0.68	3.92	2.23	54.24	38.18	36.64	11.15	27.51	5.78	22.97	16.31
6	Cyclopentane	0.25	0.22	0.28	0.05	1.27	0.48	0.28	0.03	0.44	0.28	0.62	0.25	0.39	0.13	0.58	0.15	4.34	4.63
7	n-Decane	0.29	0.77	0.38	0.00	0.38	0.00	0.38	0.00	0.93	0.58	0.32	0.07	0.30	0.16	1.78	1.33	1.71	1.02
8	2,2-Dimethylbutane	0.26	0.21	0.08	0.00	0.08	0.00	0.08	0.00	0.42	0.56	0.75	0.27	0.44	0.11	0.60	0.22	0.35	0.16
9	2,3-Dimethylbutane	0.98	1.02	0.89	0.40	1.63	1.10	0.76	0.44	3.08	1.32	2.96	1.83	1.36	0.36	2.73	1.21	7.44	5.25
10	2,3-Dimethyl pentane	0.88	0.58	0.93	0.18	1.46	0.59	0.84	0.29	0.96	0.51	1.47	0.87	0.89	0.23	1.51	0.45	2.71	1.67
11	2,3-Dimethyl pentane	0.21	0.10	0.13	0.00	0.13	0.00	0.13	0.00	0.22	0.18	0.34	0.24	0.11	0.04	0.23	0.07	0.42	0.24
12	Dodecane	1.06	0.00	1.06	0.00	0.56	0.00	0.96	0.00	0.97	0.46	0.97	0.26	0.48	0.09	1.16	0.24	1.38	0.69
13	Ethane	2.05	2.09	5.25	2.02	9.75	5.07	5.37	1.38	6.13	3.56	5.66	1.67	6.64	1.53	9.54	2.61	65.00	50.64
14	Isobutane	3.39	3.23	3.33	1.04	13.86	7.57	2.87	2.36	2.78	2.02	18.08	16.89	14.69	9.18	8.70	3.12	24.49	10.11

15	Isopentane	2.91	4.11	4.74	3.19	4.43	3.28	4.99	3.03	8.19	10.89	10.77	3.39	8.09	2.03	11.88	2.89	23.89	17.67
16	n-Octane	0.46	0.66	0.45	0.00	2.25	0.23	0.56	0.06	0.86	0.68	0.74	0.31	0.49	0.12	0.81	0.24	5.39	3.88
17	n-Pentane	2.29	1.68	2.39	0.97	10.27	7.29	2.05	0.94	4.21	5.47	4.73	1.38	3.02	0.90	4.48	1.09	32.12	26.10
18	Methylcyclohexane	0.40	0.77	0.56	0.25	1.25	0.51	0.61	0.17	0.50	0.33	0.62	0.22	0.42	0.12	0.80	0.23	7.87	6.43
19	Methylcyclopentane	0.97	0.71	1.14	0.31	1.78	0.96	0.86	0.27	1.81	1.35	2.66	1.10	1.51	0.27	2.27	0.73	8.20	5.68
20	2-Methylheptane	0.24	0.31	0.42	0.04	1.06	0.46	0.31	0.13	0.39	0.18	0.41	0.14	0.31	0.09	0.58	0.16	1.64	1.00
21	3-Methylheptane	0.26	0.22	0.28	0.06	0.62	0.11	0.28	0.09	0.38	0.18	0.39	0.11	0.20	0.04	0.42	0.12	0.84	0.50
22	2-Methylhexane	0.77	0.55	0.89	0.14	1.26	0.53	0.73	0.25	1.18	1.72	1.44	0.81	0.88	0.22	1.38	0.42	2.33	1.39
23	3-Methylhexane	0.94	1.13	0.87	0.39	3.06	1.35	0.97	0.29	1.21	0.78	0.95	0.32	0.64	0.16	1.51	0.41	11.58	9.05
24	2-Methyl pentane	1.04	1.00	0.08	0.00	3.09	1.49	0.08	0.00	4.46	3.84	3.12	1.76	1.56	0.22	3.08	0.92	7.44	5.25
25	3-Methyl pentane	1.28	1.06	0.82	0.14	1.56	0.91	1.25	0.30	1.83	1.21	4.30	1.99	1.52	0.50	2.69	1.03	6.50	3.74
26	Nonane	2.59	1.27	0.11	0.00	0.11	0.00	0.11	0.00	0.81	0.42	0.79	0.53	0.41	0.16	1.17	0.52	3.02	1.72
27	Propane	3.69	2.92	6.79	2.95	58.24	58.42	4.68	1.87	10.02	8.45	6.74	3.08	14.94	9.92	10.17	2.59	108.88	94.47
28	2,2,4-Trimethyl pentane	0.11	0.07	0.31	0.00	0.05	0.00	0.05	0.00	0.15	0.13	0.07	0.04	0.17	0.11	0.05	0.01	0.18	0.14
29	n-Undecane	1.47	1.09	0.35	0.00	0.38	0.00	0.72	0.00	1.08	0.62	0.67	0.40	0.37	0.10	1.66	1.00	1.42	0.81
	**Total alkanes**	38.00	23.39	47.98	11.88	134.60	82.64	38.51	10.21	67.47	28.89	188.20	102.20	122.60	23.56	122.80	26.71	434.94	288.00
30	1,3-Butadiene	3.18	1.67	0.46	0.26	0.46	0.07	0.39	0.21	0.05	0.00	0.58	0.50	1.14	0.47	0.90	0.18	0.53	0.37
31	Propene	0.84	1.22	2.61	0.78	8.32	8.66	1.18	0.60	2.72	5.11	3.20	1.54	5.90	0.95	5.58	1.69	2.39	1.64
32	ethyne	1.52	0.92	4.05	1.52	2.18	0.32	1.85	0.77	4.18	6.25	3.40	0.69	4.65	0.86	5.71	1.35	2.20	0.74

33	1-Butylene	1.25	1.22	1.44	0.39	7.96	4.96	1.16	0.60	2.34	1.86	10.64	7.49	7.17	2.19	5.39	1.13	4.18	3.44
34	Cis-2-butene	0.51	0.57	0.61	0.21	2.10	1.26	0.51	0.30	1.24	1.71	4.52	3.43	2.08	0.68	2.02	0.49	1.01	0.74
35	Trans-2-butene	0.57	0.96	0.79	0.21	3.23	1.94	0.54	0.30	1.10	1.16	6.74	5.32	2.73	0.91	2.65	0.63	1.41	1.00
36	Ethene	1.82	1.83	5.41	2.46	3.71	1.83	2.06	0.61	5.65	6.18	4.96	1.57	7.45	1.03	7.07	2.61	3.89	2.24
37	1-hexene	3.01	10.96	0.49	0.08	3.39	1.60	1.03	0.00	6.70	4.85	24.15	30.03	0.53	0.20	0.96	0.23	11.30	9.02
38	Isoprene	0.28	0.47	0.64	0.30	0.63	0.24	0.58	0.30	3.64	2.51	2.64	2.54	3.41	1.53	5.04	1.01	2.87	1.72
39	1-Pentene	1.14	1.03	0.86	0.85	3.98	2.54	0.61	0.59	0.72	0.61	0.59	0.25	0.75	0.42	0.96	0.23	0.58	0.41
40	Cis-2-pentene	0.51	0.76	0.62	0.40	0.40	0.17	0.34	0.22	0.62	0.64	0.63	0.27	0.64	0.14	1.34	0.35	0.77	0.72
41	Trans-2-pentene	0.46	1.12	0.53	0.45	1.91	1.11	0.29	0.25	0.56	0.68	0.47	0.28	0.81	0.27	2.25	0.58	1.12	1.05
	**Total alkenes**	15.17	12.31	19.19	6.54	38.75	22.09	11.20	4.36	30.98	2.44	63.67	32.49	38.97	6.46	41.27	7.19	35.49	10.46
42	Benzene	4.12	2.50	6.43	2.67	6.39	2.36	5.45	2.23	14.10	6.48	8.73	3.08	5.46	1.31	10.61	4.28	6.62	4.47
43	Toluene	7.65	4.14	15.60	2.68	6.53	1.41	15.82	9.31	9.25	4.08	53.04	25.33	27.75	6.50	28.66	11.30	27.79	20.53
44	Ethylbenzene	2.10	1.28	2.73	1.07	2.38	1.09	3.71	3.73	2.06	1.53	9.53	8.02	4.19	1.14	4.59	1.34	2.64	1.13
45	Phenylethylene	0.43	0.53	0.68	0.15	0.47	0.21	0.67	0.21	1.45	1.46	1.15	0.67	1.71	0.63	1.40	0.39	3.23	4.87
46	p-Xylene	1.75	0.83	2.37	0.81	1.26	0.50	3.09	2.80	1.64	1.23	4.23	2.75	2.74	0.83	3.42	1.02	2.03	1.00
47	m-Xylene	1.75	0.83	2.73	1.07	1.26	0.50	3.09	2.80	1.64	1.23	4.23	2.75	2.74	0.83	3.42	1.02	2.03	1.00
48	o-xylene	1.05	0.66	1.56	0.58	1.09	0.38	1.91	1.85	1.42	0.96	2.82	1.79	2.08	0.66	2.75	0.80	1.18	0.58
49	4-Ethyltoluene	0.31	0.54	3.20	0.50	0.71	0.08	6.29	6.51	0.05	0.00	2.37	1.97	4.27	1.76	3.50	1.10	1.19	0.82
50	1,2,4-Trimethylbenzene	0.49	0.54	1.09	0.47	0.43	0.00	5.44	4.39	1.24	0.82	0.61	0.26	1.43	0.71	1.19	0.27	0.33	0.26
51	1,3,5-Trimethylbenzene	0.30	0.54	2.20	0.36	0.32	0.00	2.80	2.10	0.44	0.43	1.25	0.84	1.40	0.67	1.39	0.30	0.38	0.27

52	Isopropylbenzene	0.23	0.17	0.80	0.00	0.80	0.00	0.50	0.00	0.19	0.13	0.67	0.60	1.68	1.09	1.07	0.60	0.11	0.06
53	1,3-Diethylbenzene	0.26	1.73	0.12	0.00	0.12	0.00	0.62	0.00	0.70	0.61	0.34	0.28	0.26	0.07	0.50	0.12	0.18	0.09
54	1,4-Diethylbenzene	0.44	1.62	0.12	0.00	0.12	0.00	0.29	0.00	0.78	0.69	0.78	0.32	0.90	0.43	0.89	0.30	0.46	0.18
55	o-Ethyltoluene	0.24	0.41	0.28	0.00	0.14	0.00	1.07	0.69	0.42	0.38	0.63	0.57	0.73	0.33	0.76	0.23	0.20	0.12
56	m-Ethyltoluene	0.40	0.38	1.48	0.18	0.32	0.00	2.04	1.34	0.65	0.48	1.48	1.20	1.32	0.52	1.41	0.41	0.43	0.32
57	n-Propylbenzene	0.23	0.25	0.38	0.00	0.12	0.00	0.71	0.33	0.32	0.27	0.74	0.85	0.46	0.23	0.52	0.14	0.15	0.09
58	1,2,3-Trimethylbenzene	0.28	0.75	0.74	0.16	0.11	0.00	1.10	0.96	0.61	0.62	0.53	0.37	0.91	0.42	1.01	0.30	0.32	0.15
	**Total aromatics**	22.03	11.70	42.51	9.85	22.57	5.87	54.60	2.27	36.98	14.41	93.14	48.64	60.06	10.87	67.07	16.52	49.25	32.61
	**Total VOCs**	75.20	40.09	109.68	23.27	195.92	103.26	104.31	46.04	135.43	46.01	345.01	170.52	221.63	34.32	231.14	46.69	519.68	309.88

ave means average concentration, s.d means standard deviation.

Table S2. Correlation coefficient (R^2) of the identified VOCs with ethyne and i-pentane for the 9 cities sampled, and benzene to toluene ratio (B/T) in 9 cities of NRC.

R^2	Shenyang		Tianjin		Anshan		Huludao		Changchun		Jilin		Harbin		Daqing	
Spceies	ethyne	Iso-pentane	ethyne	Iso-pentane	ethyne	Iso-pentane	ethyne	Iso-pentane	ethyne	Iso-pentane	ethyne	Iso-pentane	ethyne	Iso-pentane	ethyne	Iso-pentane
1,3-Butadiene	0.538	0.164	0.177	0.672	0.839	0.697	-0.188	0.42	0.725	0.758	0.245	0.77	0.664	0.608	0.759	0.879
n-Hexane	0.11	0.175	-0.297	-0.397	-0.438	-0.265	-0.042	0	0.771	0.831	0.147	-0.59	0.576	0.337	0.854	0.646
Benzene	0.387	0.125	0.871	0.704	0.519	0.043	0.156	0.588	0.85	0.885	0.252	0.554	0.332	0.455	0.857	0.424
2,2,4-Trimethyl pentane	0.579	0.29	0.057	0.571	0.853	0.77	-0.213	0.762	0.962	0.972	0.75	0.9	0.286	0.619	0.4	0.896
Toluene	0.593	0.448	0.838	0.576	-0.23	-0.215	0.435	-0.17	0.788	0.826	0.269	-0.185	0.762	0.508	0.835	0.559
Ethylbenzene	0.331	0.103	0.966	0.743	0.675	0.003	0.044	0.468	0.739	0.722	0.285	0.73	0.909	0.821	0.83	0.629
Phenylethylene	0.066	0.049	0.562	0.68	0.263	-0.435	-0.095	0.502	0.83	0.887	0.232	0.676	0.885	0.602	0.485	0.904
p-Xylene	0.219	0.195	0.964	0.772	0.767	0.136	0.148	0.431	0.797	0.843	0.171	0.712	0.926	0.711	0.832	0.623
m-Xylene	0.219	0.195	0.964	0.772			0.148	0.431	0.797	0.843	0.171	0.712	0.926	0.711	0.832	0.623
o-xylene	0.384	0.223	0.947	0.715	0.799	0.196	0.306	0.248	0.832	0.854	0.3	0.696	0.716	0.751	0.913	0.662
Cyclohexane	0.537	0.528	-0.13	-0.162	-0.403	-0.368	-0.052	0.461	0.778	0.824	0.225	-0.454	0.667	0.425	0.746	0.887
4-Ethyltoluene	0.369	0.102														
Heptane	0.319	0.477	0.662	0.531	0.336	-0.374	-0.154	0.544	0.904	0.938	0.55	0.868	0.938	0.876	0.345	0.826
Propene	0.388	0.069	0.035	0.384	0.406	0.472	-0.134	0.48	0.899	0.838	0.77	0.825	0.433	0.63	0.788	0.867
1,2,4-Trimethylbenzene	0.382	0.356														
1,3,5-Trimethylbenzene	0.378	0.097														
Iso-propylbenzene	0.06	0.136	0.939	0.598					0.751	0.745	0.531	0.476	0.38	0.685	0.884	0.768
Acetylene	-	0.117	-	0.766	-	0.732	-	-0.361	-	0.947	-	0.543	-	0.853	-	0.683

n-Butane	0.391	0.586	0.946	0.808	0.728	0.621	-0.253	0.864	0.847	0.899	0.539	0.948	0.801	0.954		
1-Butylene	0.441	0.608	0.957	0.707	0.729	0.622	-0.165	0.455	0.842	0.896	0.524	0.948	0.796	0.957	0.856	0.929
Cis-2-butene	0.558	0.443	0.749	0.972	0.918	0.669	-0.215	0.72	0.822	0.884	0.49	0.943	0.789	0.979	0.956	0.662
Trans-2-butene	0.376	0.699	0.761	0.934	0.987	0.778	-0.225	0.764	0.804	0.869	0.377	0.861	0.793	0.987	0.931	0.779
Cyclopentane	0.471	0.703	0.818	0.89	0.59	0.663	-0.099	0.092	0.927	0.863	0.849	0.76	0.939	0.942	0.306	0.796
n-Decane	0.383	0.101							0.857	0.827	0.213	0.446	0.327	0.652	0.495	0.854
1,3-Diethylbenzene	0.758	-0.263	-0.262	-0.564					0.601	0.46	0.702	0.599	0.813	0.972	0.938	0.71
1,4-Diethylbenzene	0.761	-0.123	-0.262	-0.564					0.528	0.608	0.234	0.585	0.817	0.953	0.851	0.72
2,2-Dimethylbutane	0.416	-0.098							0.977	0.951	0.448	0.558	0.914	0.928	0.705	0.293
2,3-Dimethylbutane	0.519	0.541	0.695	0.51	0.135	0.18	-0.265	0.655	0.794	0.825	0.877	0.336	0.504	0.707	0.601	0.892
2,3-Dimethyl pentane	0.617	0.452	0.766	0.713	0.837	0.267	-0.057	0.426	0.827	0.78	0.534	0.947	0.922	0.928	0.581	0.916
2,3-Dimethyl pentane	0.588	0.328							0.785	0.727	0.302	0.653	0.933	0.971	0.572	0.918
Dodecane			-0.262	-0.564			0.832	-0.52	0.83	0.733	0.637	0.697	0.775	0.948	0.509	0.868
Ethane	0.329	-0.024	0.411	0.68	0.484	0.255	-0.089	0.015	0.922	0.859	0.247	0.374	0.593	0.771	0.256	0.76
Ethene	0.467	-0.006	0.651	0.774	0.867	0.524	0.221	0.167	0.939	0.873	0.604	0.645	0.538	0.611	0.962	0.733
o-Ethyltoluene	0.384	0.031														
m-Ethyltoluene	0.455	0.151														
1-hexene	-0.23	-0.072														
Isobutane	0.66	0.237	0.888	0.936	0.662	0.122	-0.21	0.849	0.589	0.749	0.072	0.695	0.563	0.666	0.316	0.673
Isopentane	0.117	-	0.766	-	0.732	-	-0.361	-	0.947	-	0.543	-	0.853	-	0.683	-
Isoprene	0.223	0.801	0.564	0.698	0.517	0.787	0.404	-0.402	0.68	0.667	0.321	0.759	0.685	0.848	0.682	0.92
n-Octane	0.368	0.379														

n-Pentane	0.387	0.541	0.747	0.895	0.721	0.208	-0.127	0.174	0.93	0.934	0.686	0.885	0.886	0.99	0.43	0.945
1-Pentene	0.603	0.374	0.106	0.562	0.636	0.546	-0.015	-0.127	0.923	0.968	0.536	0.857	0.787	0.968	0.839	0.316
Methylcyclohexane	0.531	0.569	0.733	0.587	0.283	-0.42	-0.123	0.579	0.948	0.964	0.658	0.958	0.899	0.989	0.293	0.833
Methylcyclopentane	0.548	0.552	0.514	0.356	0.554	-0.105	-0.116	0.368	0.952	0.947	0.568	0.605	0.885	0.872	0.495	0.902
2-Methylheptane	0.623	0.448														
3-Methylheptane	0.651	0.446														
2-Methylhexane	0.697	0.465	0.758	0.706	0.88	0.387	-0.044	0.356	0.839	0.8	0.528	0.909	0.929	0.93	0.616	0.918
3-Methylhexane	0.545	0.464	0.662	0.531	0.336	-0.374	-0.154	0.544	0.959	0.958	0.645	0.968	0.911	0.973	0.311	0.835
2-Methyl pentane	0.543	0.563					0.012	-0.215	0.871	0.888	0.901	0.656	0.818	0.942	0.606	0.893
3-Methyl pentane	0.598	0.495	0.062	-0.342	-0.34	-0.184	-0.149	0.482	0.92	0.924	0.099	-0.1	0.598	0.757	0.779	0.895
Nonane	0.237	0.201							0.806	0.9	0.364	0.758	0.686	0.902	0.348	0.835
Cis-2-pentene	0.094	0.845	0.581	0.848	0.791	0.775	0.146	0.798	0.884	0.963	0.679	0.883	0.761	0.968	0.872	0.356
Trans-2-pentene	0.218	0.672	0.266	0.679	0.648	0.583	-0.043	-0.1	0.809	0.777	0.594	0.756	0.765	0.97	0.897	0.422
Propane	0.641	0.15	0.942	0.871	0.355	-0.076	-0.178	0.93	0.852	0.815	0.062	0.348	0.761	0.646	0.319	0.797
n-Propylbenzene	0.338	-0.018	0.905	0.629					0.623	0.463	-0.707	0.541	0.776	0.938	0.857	0.563
1,2,3-Trimethylbenzene	0.285	-0.004	0.993	0.841					0.823	0.818	-0.768	0.371	0.401	0.928	0.875	0.671
2,2,4-Trimethylpentane	0.627	0.04							0.861	0.897	-0.498	0.368	0.855	0.878	0.223	0.867
n-Undecane	0.031	-0.256							0.725	0.727	-0.784	0.167	0.106	0.65	0.381	0.901
B/T	0.54		0.44		0.41		0.98		0.16		0.2		0.37		0.24	

Table S3. Source apportionment of VOCs at Shenyang by PCA

	Factor				
**species**	**1**	**2**	**3**	**4**	**5**
1,3-Butadiene		0.873		0.308	
Benzene	-0.324	0.621	0.348		0.506
Toluene	-0.389	0.306	0.462		0.532
Ethylbenzene			0.826		0.403
Phenylethylene			0.942		
p,m-Xylene	0.459		0.8		
o-Xylene			0.859		
Cyclohexane					0.738
4-Ethyltoluene		0.838			
Heptane			0.889		
Propene	0.815				
1,3,5-Trimethylbenzene	0.386	0.504		-0.366	0.343
Isopropylbenzene	0.97				
Acetylene	0.963				
n-Butane	0.94				
1-Butylene			0.584	0.504	
Cis-2-Butene	0.537			0.718	
Trans-2-Butene		0.58	0.587		
n-Decane	0.899				
2,3-Dimethyl pentane		0.606		0.627	
2,3-Dimethyl pentane	0.952				
Dodecane	0.713	0.35			
Ethane	0.539	0.586			
1-Hexene	0.825				
Isobutane	0.962				
Isopentane		0.314	0.7		0.33
Isoprene				0.689	
n-Octane		0.482	0.479	0.46	-0.434
n-Pentane		0.678			
1-Pentene	0.917				
3-Methylhexane		0.702		0.493	
2-Methyl pentane				0.868	
3-Methyl pentane				0.833	
Cis-2-Pentene	0.943				
Trans-2-Pentene	0.968				

Propane		0.855			
n-Propylbenzene	0.688				-0.493
Initial eigenvalue	12.387	6.598	5.112	3.631	1.891
% of variance	33.477	17.832	13.816	9.812	5.11
Cumulative%	33.477	51.31	65.126	74.938	80.048

Table S4. Source apportionment of VOCs at Tianjin by PCA

	Factor					
**species**	**1**	**2**	**3**	**4**	**5**	**6**
1,3-Butadiene		0.934				
Benzene	0.414	0.592	0.509			
Toluene	0.447		0.549		0.399	
Ethylbenzene			0.94			
Phenylethylene			0.925			
p-Xylene			0.959			
m-Xylene			0.948			
o-xylene	0.332		0.682	0.319	0.452	
Cyclohexane	0.408		0.546		0.301	0.545
4-Ethyltoluene		0.881				
Heptane	0.704	0.432	0.485			
Propene		0.885				
1,2,4-Trimethylbenzene	0.805				0.409	
Isopropylbenzene	0.384				0.682	
Acetylene		0.898				
n-Butane	0.314					0.915
1-Butylene						0.877
Cis-2-butene				0.917		
Cyclopentane				0.856		
n-Decane	0.9					
1,4-Diethylbenzene	0.362			0.771		
2,3-Dimethyl pentane	0.61	0.471			0.349	0.323
Ethane		0.936				
Ethene		0.905				
o-Ethyltoluene	0.546				0.663	
m-Ethyltoluene	0.577	0.345	0.384		0.513	
1-hexene						0.896
Isobutane	0.77					0.567
Isopentane	0.838		0.439			
Isoprene				0.875		
n-Octane	0.68	0.461		0.365	0.327	
n-Pentane	0.924					
1-Pentene	0.55	0.355		0.614		
Methylcyclohexane	0.76	0.491				

Methylcyclopentane	0.527	0.497		0.42	0.39	
2-Methylhexane	0.886	0.303				
3-Methylhexane	0.592	0.539		0.454		
3-Methyl pentane				0.453	0.642	
Nonane	0.905					
Cis-2-pentene	0.751			0.455		
Propane		0.946				
n-Propylbenzene					0.779	
1,2,3-Trimethylbenzene	0.623		0.307		0.442	
n-Undecane	0.927					
Initial eigenvalue	19.988	7.001	4.626	3.275	2.253	1.581
% of variance	45.426	15.912	10.513	7.443	5.122	3.593
Cumulative%	45.426	61.338	71.852	79.294	84.416	88.009
